# A Novel Route to Fabricate High-Performance 3D Printed Continuous Fiber-Reinforced Thermosetting Polymer Composites

**DOI:** 10.3390/ma12091369

**Published:** 2019-04-26

**Authors:** Yueke Ming, Yugang Duan, Ben Wang, Hong Xiao, Xiaohui Zhang

**Affiliations:** State Key Lab for Manufacturing Systems Engineering, Xi’an Jiaotong University, Xi’an 710049, China; mingyueke@foxmail.com (Y.M.); xiaohongjxr@xjtu.edu.cn (H.X.); zhangxiaohui@mail.xjtu.edu.cn (X.Z.)

**Keywords:** 3D printing, continuous carbon fiber, thermosetting epoxy resin, mechanical properties

## Abstract

Recently, 3D printing of fiber-reinforced composites has gained significant research attention. However, commercial utilization is limited by the low fiber content and poor fiber–resin interface. Herein, a novel 3D printing process to fabricate continuous fiber-reinforced thermosetting polymer composites (CFRTPCs) is proposed. In brief, the proposed process is based on the viscosity–temperature characteristics of the thermosetting epoxy resin (E-20). First, the desired 3D printing filament was prepared by impregnating a 3K carbon fiber with a thermosetting matrix at 130 °C. The adhesion and support required during printing were then provided by melting the resin into a viscous state in the heating head and rapidly cooling after pulling out from the printing nozzle. Finally, a powder compression post-curing method was used to accomplish the cross-linking reaction and shape preservation. Furthermore, the 3D-printed CFRTPCs exhibited a tensile strength and tensile modulus of 1476.11 MPa and 100.28 GPa, respectively, a flexural strength and flexural modulus of 858.05 MPa and 71.95 GPa, respectively, and an interlaminar shear strength of 48.75 MPa. Owing to its high performance and low concentration of defects, the proposed printing technique shows promise in further utilization and industrialization of 3D printing for different applications.

## 1. Introduction

At present, the most commonly used polymer consumables in three-dimensional (3D) printing are thermoplastic filaments and powders, which exhibit weak load capacity, poor interlayer bonding, and low strength and hardness [1,2]. Usually, the high-performance fiber is used as reinforcement, and the polymeric resin is used as a matrix to improve the mechanical properties [3,4]. The fiber is used to enhance the corresponding strengths and moduli, whereas the resin is used to bond the fiber and uniformly distribute and transfer the external load [5,6].

**Figure 1:**
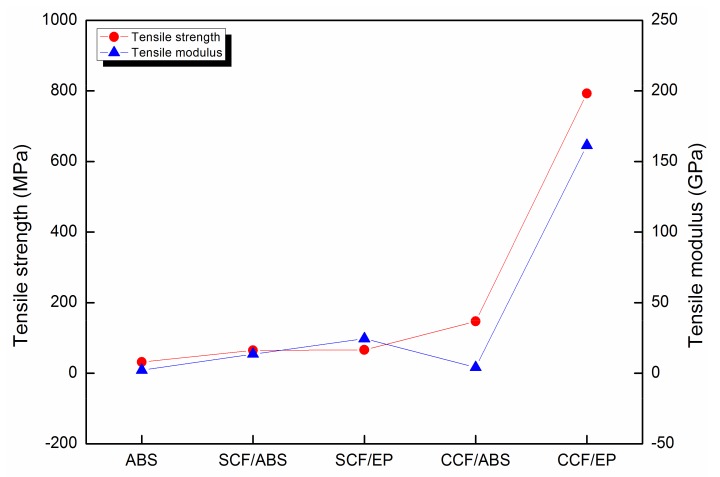
Performance evolution of 3D printing for different materials, including acrylonitrile butadiene styrene (ABS) [2], short carbon fiber (SCF)/ABS [10], SCF/epoxy resin (EP) [12], continuous carbon fiber (CCF)/ABS [13], and CCF/EP [17].

As shown in Figure 1, 3D printing of fiber-reinforced composites has achieved remarkable milestones [7,8,9]. Initially, short fibers have been added into the thermoplastic filaments of the fused filament fabrication (FFF) process to attempt to improve performance. For instance, Tekinalp et al. [10] introduced short carbon fiber (SCF) into acrylonitrile butadiene styrene (ABS) and carried out 3D printing by using FFF equipment. All printed SCF/ABS composites exhibited a fiber orientation of 91.5%. Compared with the conventional compressive molded SCF/ABS composites, the tensile strength and tensile modulus of the 3D printed SCF/ABS composites, with a 40 wt % fiber content, increased to 65.0 MPa and 13.6 GPa, respectively. Furthermore, several research efforts have been made to replace the thermoplastic resin due to its defects with a thermosetting matrix [11]. For instance, Compton et al. [12] synthesized a low-viscosity mixture by mixing several materials at a low temperature, where SCFs were used as a reinforcement, a thermosetting epoxy resin (EP) as a matrix, and an imidazole compound as a curing agent. The pre-formed samples were printed using FFF equipment. After curing, the printed SCF/EP composites with a fiber content of 35 wt % exhibited a tensile strength of 66.2 MPa. Later, continuous fiber reinforcements were used to further enhance the mechanical properties due to the ceilings of the short fiber reinforcement [13,14,15]. Yang et al. [16] utilized molten ABS to wrap the continuous carbon fiber (CCF) and carried out 3D printing by feeding, extruding, and cooling the mixture of the ABS filament. The printed CCF/ABS composites, with a 10 wt % fiber content, exhibited a tensile strength and flexural strength of 147 MPa and 127 MPa, respectively. However, the low interlaminar shear strength of 2.81 MPa reveals the vulnerable nature of the interlaminar performance due to the thermoplastic matrix. Nowadays, the continuous fiber-reinforced thermosetting polymer composites (CFRTPCs) are being used in 3D printing due to their strong intermolecular cross-linking [8]. Hao et al. [17] impregnated the continuous carbon fiber with epoxy resin (E-54). The pre-formed composites were then printed on the building platform and moved to a high-temperature chamber for curing. The as-printed CFRTPCs exhibited a tensile strength and flexural strength of 792.8 MPa and 202.0 MPa, respectively. However, Hao et al. did not discuss the detailed 3D printing issues about printing and post-curing methods.

In this study, a novel 3D printing process is proposed to solve the challenges in printing and curing. The CFRTPC composites were fabricated using the relevant equipment. The fiber content, printing precision, fiber–resin interface, and internal voids were analyzed to characterize defects, deformations, and resin distribution during impregnating, printing, and curing modules. Finally, the mechanical properties of CFRTPCs were studied by tensile, flexural, and interlaminar shear testing.

## 2. Experimental

### 2.1. Materials

The thermosetting matrix system is comprised of an epoxy resin (E-20 (D.E.R. 671), 95 wt %, Dow, Pittsburg, CA, USA), and a curing agent (dicyandiamide (DICY), 5 wt %, Yongxin plasticization, Guangzhou, China). The dual interactions of amino and cyano groups in the DICY molecules allow an extended stable pot life with epoxy groups below 150 °C [18].

The 3K carbon fiber (Tenax®-J, HTS40, 200 TEX, Toho Tenax, Co., Ltd., Tokyo, Japan) was used as reinforcement. The tensile strength and tensile modulus of the 3K carbon fiber are 4400 MPa and 240 GPa, respectively.

### 2.2. 3D Printing Process

**Figure 2:**
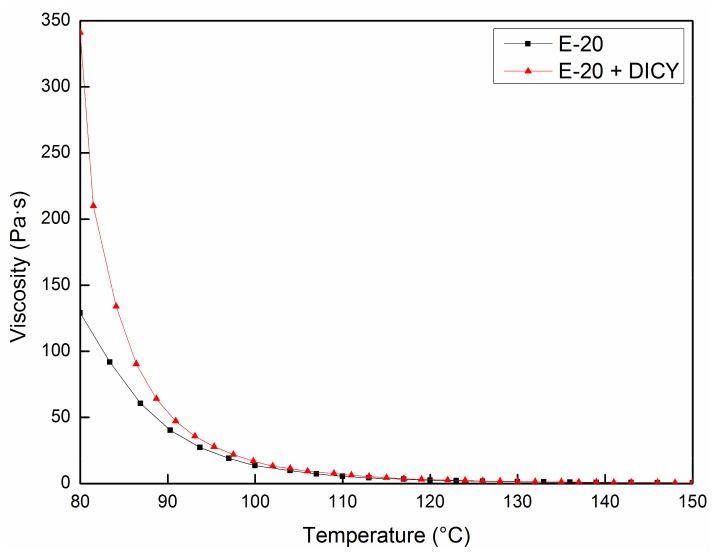
Viscosity–temperature curves of E-20 and the mixed matrix.

The high melting point (64–76 °C) allows E-20 to remain at a solid state at room temperature. The polymeric chains only possess intermolecular forces prior to the cross-linking reaction. The forces rapidly weaken and yield a low-viscosity liquid with increasing temperature, as shown in Figure 2. Therefore, the required adhesion and support during printing could be provided by using a solid-liquid resin transformation. Meanwhile, the impregnating and curing modules were separately designed to ensure fiber impregnation and to solve the problems during long-term post-curing. Hence, the whole process was separated into three modules: impregnating, printing, and curing. The low viscosity, at medium temperatures, promoted the fiber–resin interface during impregnating, whereas the high viscosity, at low temperatures, satisfied the printing requirements. Finally, a thermally initiated latent curing agent was used for curing at a higher temperature to avoid the premature cross-linking reactions between the first two modules.

#### 2.2.1. Impregnating

**Figure 3:**
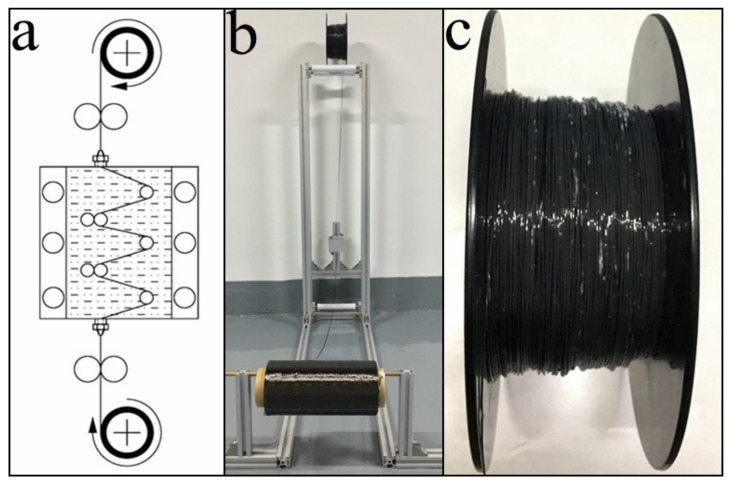
The as-designed impregnating equipment: (**a**) a schematic illustration of the impregnating process, (**b**) impregnating equipment, and (**c**) 3D printing filament of continuous fiber-reinforced thermosetting polymer composites (CFRTPCs).

As shown in Figure 3a,b, the impregnation equipment was independently designed and built. The fiber was released from the supply coil and conveyed into the molten resin tank. According to the viscosity–temperature curve of the resin matrix (Figure 2) and the minimum reaction temperature (150 °C) of DICY, the impregnating temperature was set at 130 °C. Moreover, several yarn rollers were arranged in the resin tank to ensure the penetration of resin into the fiber bundles. The multiple deflections of the impregnating path and the control of the conveying tension resulted in efficient impregnation of the fiber. The width of the fiber bundle was extended from 1.5 to 3.5 mm. The infiltrated fiber bundle was then passed through a squeezing nozzle, which scraped off the excess resin and reshaped the extended bundle into a circle. By decreasing the temperature below the melting point (64–76 °C), the resin solidified together with the fiber to form a circular bar. Finally, through rewinding, the 3D printing filament of CFRTPCs was obtained, as shown in Figure 3c.

#### 2.2.2. Printing

**Figure 4:**
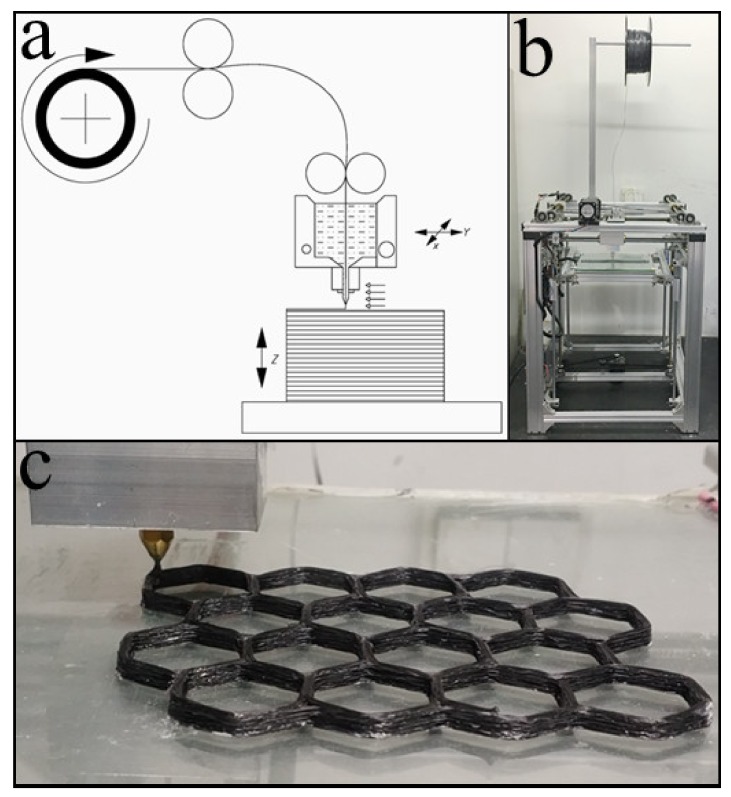
Working process of the printing module: (**a**) a schematic illustration of the printing process, (**b**) printing equipment, and (**c**) the working process of the printing nozzle.

The FFF-based printing equipment was designed and developed, as shown in Figure 4a,b. The 3D printing filament of CFRTPCs needed to pass through the heating head (130 °C) and printing nozzle in advance. The resin part of the filament was melted into a viscous state and resulted in a small molten resin tank inside the heating head. Hence, for further infiltration and subsequent printing, the filament was impregnated again due to the interactions of the heating head and the printing nozzle. After pulling out, the fiber was rapidly cooled down and attached to the platform or the former layer (Figure 4c). The heating head moved in the X-Y plane and along the cross-section contours and filling trajectories generated from the 3D model. After each layer’s generation, the platform descended along the Z-direction by a distance equal to the layer thickness. These steps were repeated until the whole pre-formed sample was printed.

#### 2.2.3. Curing

It is worth noting that the resin melts and transforms into a viscous state due to the utilization of the thermally induced curing method. Once the constraints from the glassy resin are reduced, large deformations occur at the turning points in the planned path. In combination with a negative pressure condition, the pre-formed samples are prone to collapse.

**Figure 5:**
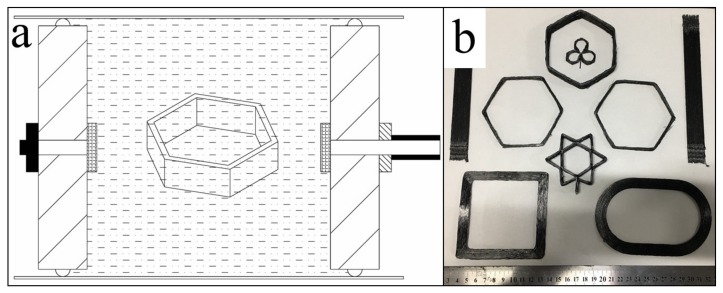
Curing module: (**a**) a schematic illustration of the curing process and (**b**) the cured CFRTPC samples.

Herein, a novel curing method is proposed to maintain the original shapes of the pre-formed CFRTPC samples and accommodate the complexity and variability of 3D printing technology (Figure 5). First, the pre-formed samples were completely buried in sodium chloride powders. Second, the pressure of −0.1 MPa was exerted by the external vacuum pump to promote the filling of internal voids and eliminate the trapped air. Then, the powders and pre-formed samples formed a dense entity to maintain the original shape. After that, the entire device was placed in an oven, infiltrating at 130 °C for 1 h and cured at 160 °C for 1 h. Finally, the CFRTPC samples were removed from the oven, rinsed with water, and dried to obtain the final products (Figure 5b).

### 2.3. Characterization

The fiber content of the 3D printing filament was calculated by thermal gravimetric analysis (TGA-DSC1, Mettler-Toledo GmbH, Greifensee, Schweiz). The surface of the impregnated filament and the 3D profile of the printed fiber bundle were scanned by laser scanning confocal microscopy (OLS4000, Olympus, Tokyo, Japan). The fiber–resin interface and resin distribution in the pre-formed and cured samples were observed with a scanning electron microscope (SEM, S-3000N, Hitachi, Tokyo, Japan) at an acceleration voltage of 30.0 kV. The corresponding internal structure and void distribution were obtained by the micron X-ray 3D imaging system (YXLON international GmbH, Hamburg, Germany) with an accelerating voltage of 80.0 kV and an image resolution of 8.0 μm.

**Figure 6:**
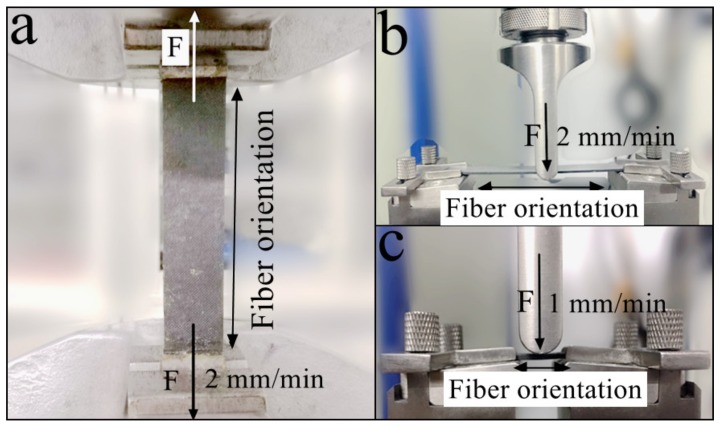
The specimen dimensions and operational specifications during (**a**) the tensile test, (**b**) the three-point bending test, and (**c**) the interlaminar shear test.

Finally, the mechanical characterization was carried out using the electromechanical universal testing machine (MTS Systems, Co., Ltd, Shenzhen, China) to obtain the mechanical properties of the cured CFRTPCs. The experimental apparatus and operation specifications are shown in Figure 6. Moreover, five independently carried out measurements were averaged out for each property.

#### 2.3.1. The Tensile Test

As shown in Figure 6a, the tensile test was conducted according to the ISO 527:1997 standard (plastics–determination of tensile properties). The dimensions of the tensile specimen were 250 × 25 × 2 mm. The fiber was unidirectionally printed at 0°, corresponding to the longest side of the specimen and force loading direction. The loading rate was 2 mm/min.

#### 2.3.2. The Three-Point Bending Test

As shown in Figure 6b, the flexural test was conducted according to the ISO 14125:1998 standard (fiber-reinforced plastic composites–determination of flexural properties). The dimensions of the flexural specimen were 100 × 15 × 2 mm. The fiber was unidirectionally printed at 0°, which corresponds to the longest side of the specimen. The vertical force was applied at mid-point of the longitudinal direction, and the loading rate was 2 mm/min.

#### 2.3.3. The Interlaminar Shear Test

As shown in Figure 6c, the interlaminar shear test was conducted according to the ISO 14130:1997 standard (fiber-reinforced plastic composites–determination of apparent interlaminar shear strength by short-beam method). The dimensions of the shear specimen were 20 × 10 × 2 mm. The fiber was printed unidirectionally at 0°, which corresponds to the longest side of the specimen. The vertical force was applied at the mid-point of the longitudinal direction, and the loading rate was 1 mm/min.

## 3. Results and Discussion

### 3.1. Fiber Content

The fiber content in CFRTPCs was mainly controlled by the impregnating module. After impregnating with the molten matrix, the continuous fiber bundle was extruded through a squeezing nozzle to scrape off the excess resin. The amount of the scraped resin can be controlled by adjusting the nozzle diameter.

**Figure 7:**
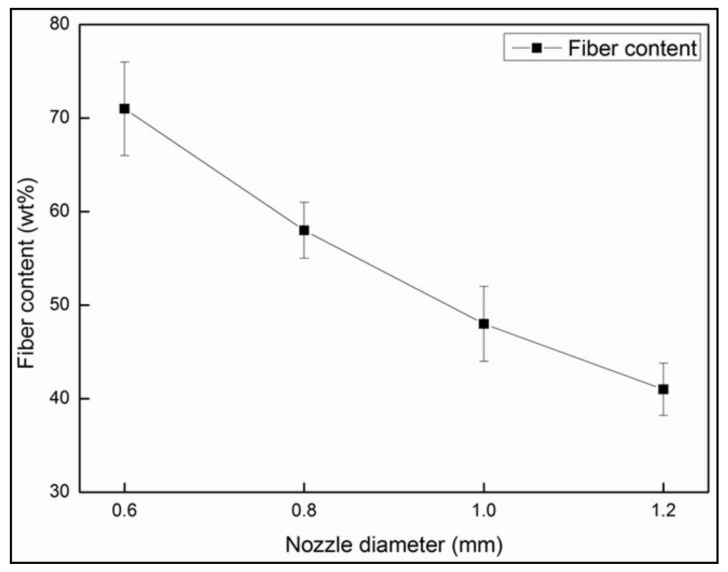
The influence of nozzle diameter on the fiber content in CFRTPCs.

**Figure 8:**
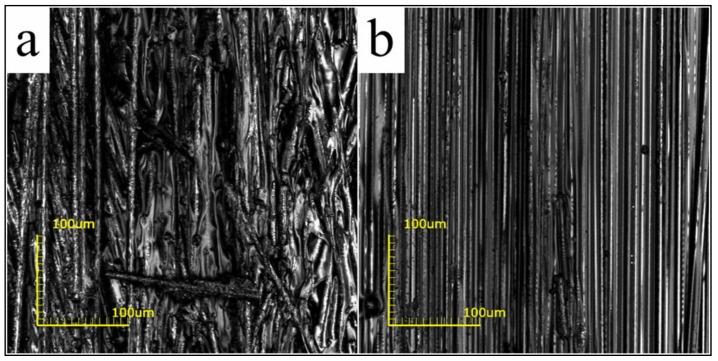
The surface of the filaments impregnated by (**a**) 0.6 mm and (**b**) 1.0 mm diameter nozzles.

As more resin was scraped off, the fiber content significantly increased with decreasing nozzle diameter, as shown in Figure 7. The highest fiber content of 71.05 wt % was achieved at a nozzle diameter of 0.6 mm. However, the damage to the fiber surface was exacerbated with the shrinkage of the nozzle diameter. The surface of the filament, impregnated by 1.0 mm and 0.6 mm nozzles, is presented in Figure 8. The smaller diameter and the sharp metal edges resulted in a large amount of fiber breakage and curling (Figure 8a). One should note that these defects inevitably influenced the mechanical properties of the obtained CFRTPCs by the subsequent printing and curing modules. The broken fibers were unable to carry and transfer stress during tensile and compressive processes, whereas the curled fibers caused void defects, which acted as crack sources and led to inferior interlayer performance. On the other hand, an excessively large nozzle diameter resulted in low fiber content and uneven resin distribution, which is also not desirable from an application viewpoint. Based on these observations, the squeezing nozzle diameter was set at 1.0 mm. The surface of the corresponding 3D printing filament, with a fiber content of 48.33 wt % and a diameter of 1.0 mm, is shown in Figure 8b.

### 3.2. Printing Precision

**Figure 9:**
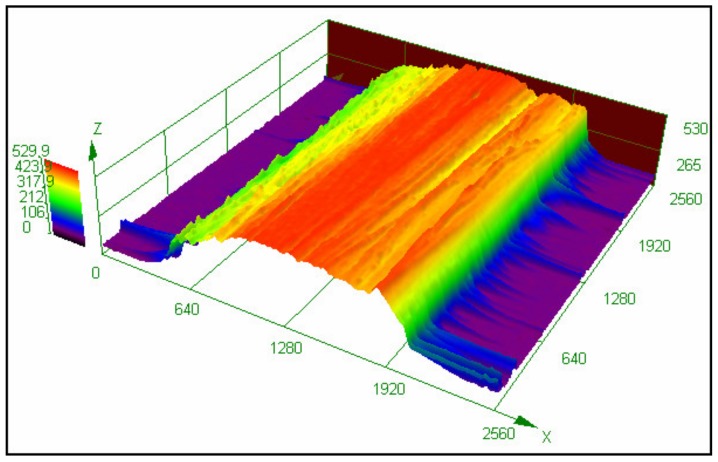
3D profile of the printed fiber bundle with a width of 1.8 mm and a thickness of 0.4 mm.

Based on the diameter of the impregnated filament, the diameter of the printing nozzle was determined to be 1.0 mm. During printing, the filament transforms into a viscous state inside the heating head (130 °C), which is soft and can be reshaped. After pulling out, the filament was flattened from a circular cross section into a rectangular shape by the printing nozzle, cooled down, and attached to the platform. The 3D profile of the printed fiber bundle is presented in Figure 9. The average width and thickness of the cross section are 1.8 mm and 0.4 mm, respectively.

**Figure 10:**
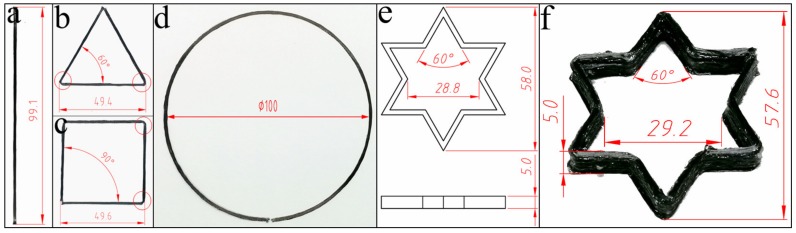
The printing tests and corresponding dimensions of (**a**) line, (**b**) triangle, (**c**) quad, and (**d**) circle and the comparison between the dimensional differences in the (**e**) original 3D model and (**f**) printed pre-formed sample.

A suitable printing speed, matching with the adhesion method of the resin’s solid–liquid transition, renders an excellent printing quality and accuracy. Hence, a constant printing speed of 10 mm/s was selected, and repeated printing tests were carried out using lines, triangles, quads, and circles to measure the dimensional error from the planned paths. As shown in Figure 10a, the printing of the lines was slightly shorter due to the poor positioning accuracy of the large printing nozzle, which can be adjusted by code compensation. In addition, the fiber was dragged up from the corners, as shown in Figure 10b,c. The driving force during printing was mainly provided by the adhesion of the solid–liquid resin transition and the traction from the printed fiber bundle. The adhesion was always the same due to the constant temperature difference before and after printing. However, the traction had to be along the printing direction due to the vector characteristics, and it might cause a printing error when the curvature suddenly changes. Therefore, in the case of large angle deflections, it was necessary to reduce the printing speed and lower the nozzle height to increase the compaction force. Finally, during arc printing with constant curvature, as shown in Figure 10d, the fiber bundle rendered a uniform width with high precision.

Furthermore, a hexagonal star was selected to analyze the printing precision in the case of the Z-direction and complex structures. Comparing the printed pre-formed sample with the original 3D model (Figure 10e,f), the dimensional differences mainly appeared at the corner positions and contributed to the error in longitudinal and transverse directions. However, the other dimensions, such as height and angle, were well controlled because those were mainly determined by the settings of X–Y–Z motion distance and direction. The shape and size remained the same as the original 3D model. The overall dimensional error was less than 2%.

### 3.3. Interface and Voids

Considering the high-temperature stability, easy removal, and cost, sodium chloride (NaCl) was used as the final powder in the curing module. The diameter of the sodium chloride particles ranged from 10 to 50 μm. A flexible peel ply was used to wrap the pre-formed sample to prevent the embedment of these particles inside the sample during curing. The pressure difference due to the externally applied vacuum filled the gaps in the pre-formed sample and maintained the original shape. Finally, the high temperature activates the DICY and led to a cross-linking reaction.

**Figure 11:**
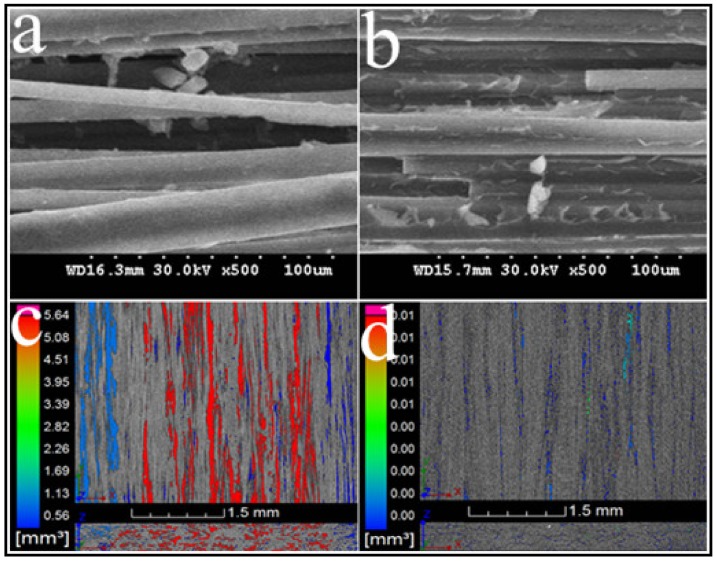
Fiber–resin interface and internal voids of the (**a**,**c**) pre-formed and (**b**,**d**) cured samples, respectively.

Figure 11 shows that the proposed curing method effectively improves the fiber–resin interface. The tightly squeezed particles resulted in a compact structure. In the case of the pre-formed sample (Figure 11a,c), the surface of the fiber bundle was partially wrapped with resin, and dense voids were observed. One should note that the DICY, with a melting point of 208–211 °C, just scattered within the gaps of the fiber in the form of solid particles. Moreover, the void content in the pre-formed sample was ~10.05%. On the other hand, E-20 and DICY melted and formed a molten mixture with low viscosity (Figure 2) due to the infiltration at 130 °C for 1 h before curing. Under the influence of a negative pressure, the molten mixture moved and filled the inner voids. The hydrogen atoms on the amines in DICY then initiated an open ring reaction with the epoxy group during subsequent curing at 160 °C for 1 h. In addition, the nitrile group reacted with the hydroxyl group to produce amides, which further reacted with the epoxy group [18,19]. After diffusion, E-20 was continuously consumed until the cross-linking reaction was completed. Therefore, in the case of cured samples, the surface of the fiber was fully and evenly covered with resin (Figure 11b) and the internal voids were also well filled (Figure 11d). The void content was reduced from 10.05 to 2.53%. The degree of cross-linking reaction was over 99%, as measured by differential scanning calorimetry.

### 3.4. Mechanical Characterization

#### 3.4.1. Tensile Test Results

**Figure 12:**
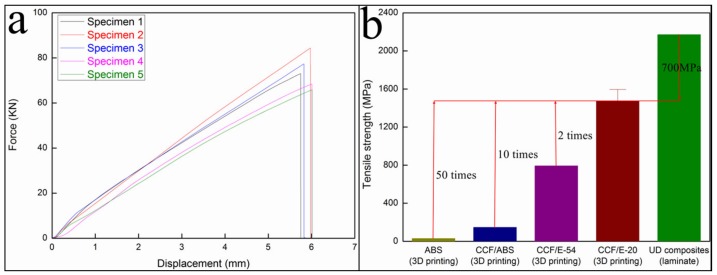
Tensile characterization: (**a**) the force–displacement curves and (**b**) tensile strengths of different materials.

Figure 12a shows that the tensile strength and tensile modulus of the cured CFRTPC samples were 1476.11 MPa and 100.28 GPa, respectively. The comparison of different materials (Figure 12b) reveals that the obtained tensile strength was twice as high as that of the 3D-printed CCF/E-54 (792.8 MPa) [17] and ~70% of the unidirectional (UD) composites (laminate, 2171.85 MPa) [20].

#### 3.4.2. Three-Point Bending Test Results

**Figure 13:**
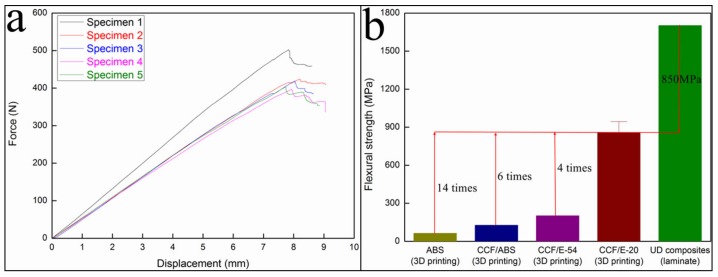
Flexural characterization: (**a**) the force-displacement curves and (**b**) flexural strengths of different materials.

As shown in Figure 13a, the flexural strength and flexural modulus of the cured CFRTPC samples were 858.05 MPa and 71.95 GPa, respectively. The comparison of different materials (Figure 13b) reveals that the obtained flexural strength was ~4 times higher than that of the 3D-printed CCF/E-54 (202.0 MPa) [17] and close to the UD composites (laminate, 1703.01 MPa) [20].

#### 3.4.3. Interlaminar Shear Test Results

The interlaminar shear strength of the cured CFRTPC samples was increased to be 48.75 MPa, but it was less than half of that of the UD composites (106.87 MPa, laminate) [20].

These results indicate that the mechanical properties, such as the tensile and flexural strengths and moduli, of the 3D-printed CFRTPCs were remarkably improved. One should note that a higher fiber content was obtained due to the introduction of the impregnating module. Moreover, the fiber and resin were evenly distributed, and an extensive cross-linking between the polymer chains of the thermosetting matrix was achieved due to the infiltration and curing processes. It is worth mentioning that a firm polymeric network can effectively carry and transfer the external load. However, the interlaminar shear strength of the 3D-printed CFRTPCs was quite low. Owing to the absence of any subsequent rolling or compaction process after the printing of each layer, the layer thickness was mainly determined by the Z-axis distance, which resulted in insufficient connectivity between layers. Hence, weak interlaminar bonding is one of the most vulnerable aspects of 3D-printed CFRTPCs.

### 3.5. Fracture Analysis

#### 3.5.1. Tensile Fracture Mode

**Figure 14:**
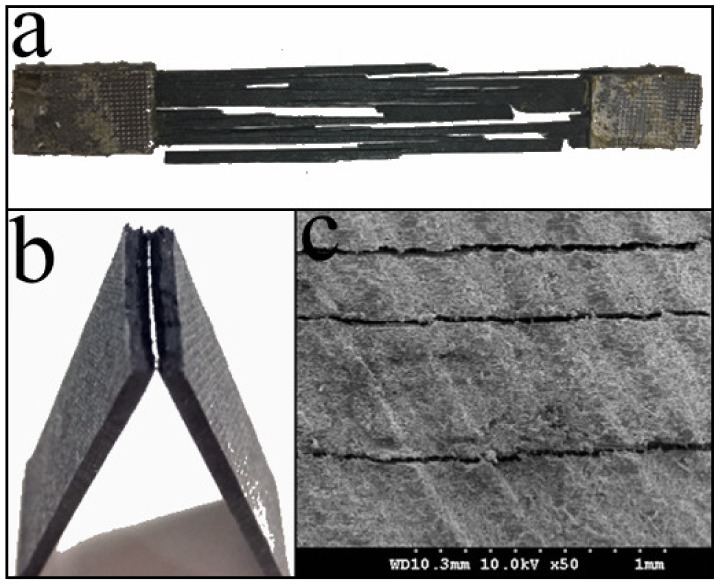
Fracture modes of the 3D-printed CCF/E-20 after (**a**) the tensile test, (**b**) the three-point bending test, and (**c**) the interlaminar shear test.

As shown in Figure 14a, the tensile fracture mode of the 3D-printed CCF/E-20 sample was fiber splitting. The whole sample was split into several strips along the printing direction. The multiple parts after the fracture indicate that there were voids and cracking defects inside the sample. Meanwhile, the same direction of fiber splitting also suggests that the bonding between the fiber bundles was not enough along the printing direction. The scratches, caused by the deflection of the fiber bundle at the exit of the printing nozzle, resulted in a higher amount of fiber damage. Therefore, when the load exceeded the bearing capacity limit, the sample split into multiple strips along the printing direction. One should note that the observed fracture mode was better than the fiber extraction from the resin of 3D-printed CCF/ABS [16], but it was still not as good as the regular brittle fracture of UD composites (laminate) [21], which suggests that the tensile properties can be further enhanced.

#### 3.5.2. Flexural Fracture Mode

As shown in Figure 14b, the flexural fracture mode of 3D-printed CCF/E-20 sample was brittle rupture. The fractured part of the 3D-printed CCF/E-20 sample was divided into two regions, referred to as the flat compression fracture zone and the coarse tensile fracture zone, due to the dual influence of tensile and compressive stresses during bending. In the perpendicular direction, the fiber was tightly bonded to the resin and formed a strong fiber–resin interface, which implies that the external load can be effectively transferred and distributed.

#### 3.5.3. Shear Fracture Mode

In the case of interlaminar shear analysis, the 3D-printed CCF/E-20 sample exhibited a multi-layer shear failure, as shown in Figure 14c. The test specimen was subjected to a diagonal triangular stress distribution, which resulted in a stress gradient between the layers [22]. Unlike 3D-printed CCF/ABS [16], the fiber did not peel off from the resin, but the presence of multiple shearing surfaces and a low interlaminar shear strength of 48.75 MPa reveal that the interlayer performance of 3D-printed CCF/E-20 was not satisfactory.

## 4. Conclusions

In summary, a novel 3D printing technology for CFRTPCs was investigated in detail. The main conclusions are summarized below:

1. The whole fabrication process was separated into three independent modules: impregnating, printing, and curing. The required experimental equipment for each module was independently designed and developed.
2. A high fiber content of 48.33 wt % was achieved through impregnation. The dimensional precision was obtained by optimizing the printing process. The overall dimensional error was less than 2%. After curing, the fiber–resin interface was significantly improved, and the void content was reduced from 10.05 to 2.53%.
3. The mechanical properties of the 3D-printed CFRTPCs were evaluated by tensile, flexural, and interlaminar shear testing. The results reveal that the tensile strength and tensile modulus were 1476.11 MPa and 100.28 GPa, respectively; the flexural strength and flexural modulus were 858.05 MPa and 71.95 GPa, respectively; and the interlaminar shear strength was 48.75 MPa. Finally, the fracture analysis demonstrated the brittle nature of CFRTPCs and the inferior interlayer performance.
